# 16S rRNA Gene Amplicon Sequence Data from Feces of Five Species of Wild Animals in Japan

**DOI:** 10.1128/MRA.00368-20

**Published:** 2020-05-28

**Authors:** Kasumi Ishida-Kuroki, Nachiko Takeshita, Yoshihiro Nitta, Takehisa Chuma, Ken Maeda, Hiroshi Shimoda, Ai Takano, Tsutomu Sekizaki

**Affiliations:** a Research Center for Food Safety, Graduate School of Agricultural and Life Sciences, The University of Tokyo, Tokyo, Japan; b Laboratory of Veterinary Public Health, Joint Faculty of Veterinary Medicine, Kagoshima University, Kagoshima, Japan; c Laboratory of Veterinary Microbiology, Joint Faculty of Veterinary Medicine, Yamaguchi University, Yamaguchi, Japan; d Laboratory of Veterinary Epidemiology, Joint Faculty of Veterinary Medicine, Yamaguchi University, Yamaguchi, Japan; Indiana University, Bloomington

## Abstract

We report 16S rRNA amplicon sequence data from feces from 58 wild boars, 60 feral raccoons, 9 wild Japanese badgers, 21 wild masked palm civets, and 8 wild raccoon dogs in Japan. The predominant bacterial taxa in the fecal microbiota were similar in part but varied among the animal species.

## ANNOUNCEMENT

Gut microbiota of animals are affected by host diet and phylogeny (1, 2). Comparative analyses of microbiota in feces of domestic pigs (Sus scrofa *domesticus*) and wild boars (Sus scrofa) have shown differences in the abundance of bacterial taxa (3). However, information on the microbiota of other wildlife is limited (4). In this study, we collected feces from five species of omnivorous wild mammals, including wild boars, in Japan and analyzed the microbiota.

The populations of some wild animals in Japan have rapidly increased in the past 2 decades. Some of these animals are considered to be harmful and are subjected to pest control. In particular, open hunting of wild boars is permitted in the winter season, and some local governments have now established control over these animals in all seasons. Feral raccoon (*Procyon lotor*) is an alien species, and their extermination has been implemented under the regulation of local governments.

Fifty-eight wild boars were killed by licensed hunters in Yamaguchi and Kagoshima prefectures in 2014 to 2019. The feces were collected from aseptically removed rectums and placed in sterilized tubes. Rectal feces from 60 feral raccoons, 9 wild Japanese badgers (*Meles meles anakuma*), 21 wild masked palm civets (*Paguma larvata*), and 8 wild raccoon dogs (*Nyctereutes procyonoides*), which were either trapped for control or died due to traffic accidents or fungal infection, were collected, as described above, by the Hikiiwa Park Center in Wakayama Prefecture in 2019. All samples were immediately cooled and stored at −20°C until use. Frozen samples were thawed and centrifuged at 13,000 × g for 5 min at 4°C. The pellet was washed twice with sterile 0.85% saline, and DNA was extracted from samples using the PowerFecal DNA isolation kit (Qiagen, Hilden, Germany). Zirconia beads (Toray, Tokyo, Japan) were used for the efficient destruction of bacterial cells, as described previously (5).

The V3 to V4 regions of 16S rRNA genes in the extracted DNA were amplified with primers 341F (5′-ACACTCTTTCCCTACACGACGCTCTTCCGATCT-NNNNN-CCTACGGGNGGCWGCAG-3′) and 805R (5′-GTGACTGGAGTTCAGACGTGTGCTCTTCCGATCT-NNNNN-GACTACHVGGGTATCTAATCC-3′) (6), including an overhang adapter sequence (Illumina, San Diego, CA, USA). Sequencing was performed using the 2 × 300-bp paired-end method on the MiSeq platform with a MiSeq v3 reagent kit (Illumina) (7). The IM-TORNADO pipeline (v2.0.3.2) (8) was used for processing FASTQ reads with default parameters except for Trimmomatic (leading, 20; trailing, 20; minlen, 180) (7). The reads were filtered for quality using Trimmomatic and merged using scripts in the pipeline. A total of 14,482 to 87,238 high-quality reads were obtained from each sample (Table 1). The pipeline used mothur (9) for operational taxonomic unit (OTU) clustering at 100% sequence identity and a k-mer-based approach for taxonomic assignment using the Ribosomal Database Project (RDP) naive Bayesian classifier (10) with a threshold of 80% bootstrap confidence. Each OTU was assigned at the family level against the RDP database (11) at 97% sequence identity. The study was confirmed to not require approval under the code of ethics for animal experiments by the Animal Research Committee of the University of Tokyo.

**TABLE 1:** Summary of samples and sequence data

Animal	Accession no.	Sampling location(s) (GPS coordinates)	No. of raw reads (mean ± SD)	No. of high-quality reads (mean ± SD)	Top three families (avg relative abundance [%])		
					First	Second	Third
Wild boars	DRR220796 to DRR220853	Shimonoseki, Yamaguchi, Japan (34°15'N, 131°01'E); Kagoshima, Japan (31°19'N, 130°54'E^*a*^)	43,391 ± 5,383	21,307 ± 3,263	*Ruminococcaceae* (16.9)	*Lachnospiraceae* (12.7)	*Prevotellaceae* (9.9)
Feral raccoons	DRR220854 to DRR220913	Tanabe, Wakayama, Japan (33°49'N, 135°34'E); Nishimuro, Wakayama, Japan (33°37'N, 135°30'E); Hidaka, Wakayama, Japan (33°54'N, 135°16'E)	51,616 ± 16,487	32,798 ± 10,990	*Clostridiaceae* (22.6)	*Peptostreptococcaceae* (18.9)	*Enterobacteriaceae* (16.8)
Japanese badgers	DRR220914 to DRR220922	Nishimuro, Wakayama, Japan (33°37'N, 135°30'E)	43,724 ± 2,176	27,117 ± 2,774	*Enterobacteriaceae* (24.8)	*Clostridiaceae* (22.8)	*Peptostreptococcaceae* (20.6)
Masked palm civets	DRR220923 to DRR220943	Tanabe, Wakayama, Japan (33°49'N, 135°34'E); Nishimuro, Wakayama, Japan (33°37'N, 135°30'E); Hidaka, Wakayama, Japan (33°54'N, 135°16'E)	41,439 ± 5,304	27,155 ± 3,593	*Lachnospiraceae* (25.5)	*Streptococcaceae* (16.8)	*Enterobacteriaceae* (15.7)
Raccoon dogs	DRR220944 to DRR220951	Tanabe, Wakayama, Japan (33°49'N, 135°34'E); Nishimuro, Wakayama, Japan (33°37'N, 135°30'E)	46,115 ± 4,606	21,272 ± 3,186	*Lachnospiraceae* (24.7)	*Prevotellaceae* (12.5)	*Fusobacteriaceae* (11.1)

a Approximate position is shown because of an agreement with hunters.

Taxonomic classification at the family level showed that the bacterial taxa *Lachnospiraceae*, *Peptostreptococcaceae*, and *Clostridiaceae* were ranked in the top 8 bacterial taxa for all five omnivores. The top 10 bacterial taxa were the same for the two alien species (raccoons and masked palm civets) but differed in abundance. The predominant bacterial taxa in wild boars were almost the same as those described previously (3) but differed in abundance, i.e., *Ruminococcaceae* (16.9%), *Lachnospiraceae* (12.7%), and *Prevotellaceae* (9.9%) (Table 1). The predominant bacterial taxa in raccoons were *Clostridiaceae* (22.6%), *Peptostreptococcaceae* (18.9%), and *Enterobacteriaceae* (16.8%), which were the same as those in Japanese badgers, i.e., *Enterobacteriaceae* (24.8%), *Clostridiaceae* (22.8%), and *Peptostreptococcaceae* (20.6%) (Table 1). The predominant bacterial taxa in masked palm civets were *Lachnospiraceae* (25.5%), *Streptococcaceae* (16.8%), and *Enterobacteriaceae* (15.7%), while *Lachnospiraceae* (24.7%), *Prevotellaceae* (12.5%), and *Fusobacteriaceae* (11.1%) were predominant in raccoon dogs (Table 1).

### Data availability.

Data sets generated by 16S rRNA gene amplicon sequencing in this study have been deposited in the DNA Data Bank of Japan (DDBJ)/Sequence Read Archive (SRA) under accession numbers DRA009965, DRA009966, DRA009967, DRA009968, and DRA009969 for wild boars, feral raccoons, wild Japanese badgers, wild masked palm civets, and wild raccoon dogs, respectively.
